# Porous ground treatments for propeller noise reduction in ground effect

**DOI:** 10.1038/s41598-024-82876-9

**Published:** 2025-01-22

**Authors:** Hasan Kamliya Jawahar, Liam Hanson, Md. Zishan Akhter, Mahdi Azarpeyvand

**Affiliations:** 1 Department of Aerospace Engineering, University of Bristol, Bristol, BS8 1TR UK; 2 Renewable & Sustainable Energy Research Center, Technology Innovation Institute, Abu Dhabi, 9639 UAE

**Keywords:** Aeroacoustics, Propeller noise, Porous materials, Ground effect, Urban Air Mobility, Aerospace engineering, Mechanical engineering

## Abstract

This study investigates the aerodynamic and aeroacoustic behavior of propellers operating in ground-effect conditions, with an emphasis on the impact of porous ground surface treatments. The investigation explores the potential of porous materials to reduce propeller noise near the ground, a major barrier to the acceptance and integration of Urban Air Mobility (UAM) systems. Experiments were conducted in an anechoic chamber using an APC $10 \times 5.5$ inch propeller in a pusher configuration. The setup used a rigid flat plate to act as the ground plane at various distances from the propeller. The ground plane was treated with three types of porous foams, each with different pore densities and thicknesses. Noise measurements were taken using a polar array of microphones positioned in both near-field and far-field locations. The results show that porous surface treatments significantly enhance noise suppression. Coherence analysis revealed that porous treatments improve the spatial consistency of acoustic signals, making noise propagation more predictable and controllable. The study also highlights that the interaction between wake flow and porous surfaces leads to greater noise suppression and stability in the hydrodynamic pressure field. These findings have significant implications for designing quieter, more efficient UAM vehicles, aiding their integration into urban environments.

## Introduction

The world is becoming increasingly interconnected culturally, economically, and politically through globalisation, driving urbanisation as populations concentrate in global cities ^1,2^. This rapid urbanisation places significant strain on infrastructure, particularly transport systems, due to rising demand for road traffic between and within urban areas ^3^. Urban Air Mobility (UAM) presents a promising solution to many of the challenges posed by road-based transportation systems ^4–6^.

UAM involves transporting cargo and passengers within urban environments using small and medium Unmanned Aircraft Systems (UAS), including electric Vertical Takeoff and Landing (eVTOL) and electric Conventional Takeoff and Landing (eCTOL) aircraft ^7^. These vehicles feature unconventional layouts with new propulsion systems enabling vertical takeoff and landing in dense urban environments. Most concepts rely on rotors and propellers ^7,8^, powered by hybrid or fully electric systems ^6,9,10^. However, noise is a significant barrier to UAM adoption, especially given the frequent, low-altitude flights over populated areas ^7^. With current battery limitations and the need for extended operational ranges ^11^, understanding the aerodynamic performance of these configurations across flight phases is critical.

While many efforts have been made in understanding the aerodynamic and aeroacoustic performance of smaller-scale propellers synonymous with UAS or eVTOL concepts^12–17^, much of the focus has been on the two main stages of flight: hover and forward flight. Surprisingly little effort has been dedicated to understanding the noise emissions in the take-off and landing phases of operation and the interactions of the propellers with the ground.

When propellers operate within close proximity to the ground, aerodynamic perturbations occur, affecting the performance and stability of the aircraft^18^. This phenomenon, known as Ground Effect (GE), occurs as the propeller wake expands near the ground surface, transitioning from vertical downwash to radial outwash parallel to the surface. GE affects the thrust and power of the rotor, influencing flight stability when aircraft are close to ground^19–26^.

**Fig. 1:**
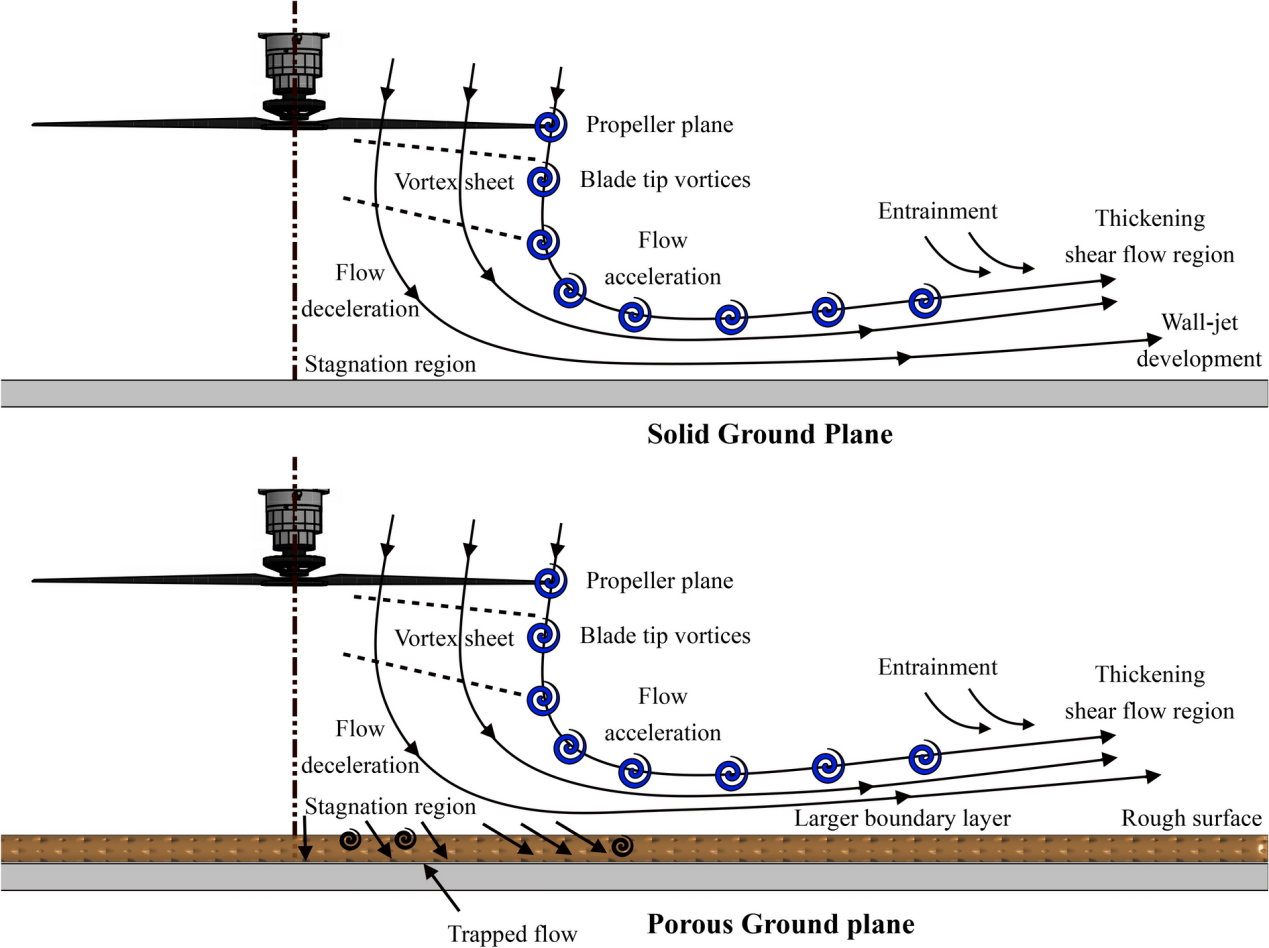
General flow development of propeller outwash subjected to solid and porous ground plane (GP) configurations.

Figure 1 illustrates the flow characteristics of a single propeller operating under GE conditions. GE is further categorized into: in ground effect (IGE) and, out of Ground Effect (OGE). IGE occurs when the propeller’s plane of rotation is within one propeller diameter from the ground, while OGE refers to the condition where the propeller is positioned further away from the ground. In OGE, the wake remains symmetrical with tip vortices contracting toward the rotational axis before mixing into the turbulent far wake. Conversely, in IGE, the downwash expands radially along the ground plane, with tip vortices deforming and interacting with the wall-jet shear layer, leading to aperiodic behavior and instabilities^27–29^.

GE is significant in various scenarios where UAM operate near flat surfaces such as ceilings^30^, walls^31,32^, and confined flight environments^28,33^. While GE has been well-studied for manned helicopters with flat-blade rotors and pitch control^27,34–37^, its impact on UAS and eVTOL aircraft, which typically use small fixed-pitch propellers, remains underexplored^38^.

Characterisation of the aerodynamic performance of propellers in both IGE and OGE has mainly been evaluated experimentally^19,22,28,32,33^. While low-fidelity numerical modelling techniques such as Blade Element Momentum Theory (BEMT) exist for aerodynamic performance prediction of propellers, GE is not commonly included due to the complexity of modelling aside from a single study by Eberhart^38^. A classical analytical model of GE was proposed by Cheeseman et al.^34^ which utilised potential flow with a single source to model the rotor airflow and the method of images to account for the GE. Empirical expressions for the rotor thrust in GE for small UAV^39^ and large helicopters^40^ have been explored in literature.

Existing literature often analyses GE through the lens of changes in thrust and power ratio. Betz^41^ set the precedent of representing thrust and power as ratios of IGE to OGE values. Numerous studies have established that propellers enter GE at a vertical distance equal to the blade diameter^38,42,43^. To the author’s best knowledge, aeroacoustic characterisation of propellers within GE and potential noise mitigation strategies of propellers IGE remain unexplored^44^.

A prior investigation by the authors^29^ comprehensively explored the aerodynamic and aeroacoustic characteristics of a two-bladed propeller in a pusher configuration within GE, revealing significant increases in thrust, torque, and power coefficients. The introduction of the ground plane (GP) notably amplified broadband noise spectra, highlighting the role of acoustic reflection and identifying shielded and reflection zones, crucial for noise mitigation strategies. Building on this foundation, the current study applies porous treatments to the solid ground in GE, aiming to enhance aeroacoustic performance by disrupting airflow, dampening the recirculation, turbulence ingestion, and therefore influencing noise propagation. This research seeks to uncover innovative approaches for noise reduction and aerodynamic optimization in vertical flight, contributing to sustainable and acoustically efficient UAM solutions. The work aligns with the goals of the EU Horizon Europe Framework Programme and supports Sustainable Development Goal (SDG) 11 (Sustainable Cities and Communities) and SDG 9 (Industry, Innovation, and Infrastructure).

Building on the foundational aspects of this study, an intriguing facet of porous media in noise mitigation is the role of natural vegetation. Notably, natural vegetation demonstrates significant noise-absorbing qualities^45^. The insights gleaned from our current research could extend to the design and strategic implementation of rooftop gardens in urban environments. Such green spaces, acting as natural porous media, have the potential to substantially attenuate UAM operational noise, concurrently contributing to pollution reduction and thermal regulation of buildings. This perspective is in line with the broader objectives of fostering sustainable and greener urban spaces, enhancing the livability of cities while integrating advanced mobility solutions. The potential integration of these natural elements into urban planning and UAM infrastructure design not only addresses environmental and societal challenges but also enriches the urban landscape, promoting a holistic approach to urban air mobility integration.

This study presents a detailed aerodynamic and aeroacoustic analysis of an isolated propeller operating GE with a typical Solid ground along with various surface treatments using porous materials. The main purpose of this study is to parametrically characterise the aerodynamic and aeroacoustic performance of propellers in varying levels of GE while evaluating the impacts of various porous treatments. The paper is organised as follows: Section 2 describes the experimental setup, the propeller being tested and the porous treatments explored. Section 3 reports on the aerodynamic performance of the propeller in various levels of GE, the untreated ground case and different porous cases are explored in terms of the thrust and power coefficients. Further it consists of detailed aeroacoustic results and analysis with both the far-field and near-field spectra, overall sound pressure level (OASPL) and coherence plotted. Finally, Section 4 summarises the work undertaken and concludes the main points to be taken from this body of research. Based on the author’s literature review, this study is the first to investigate the aerodynamic and aeroacoustic performance of an isolated propeller in ground effect (GE) with various porous ground treatments. It provides new insights into how these surface modifications influence propeller performance and noise, contributing foundational knowledge for Urban Air Mobility (UAM) applications.

## Experimental setup

Experiments to investigate propellers operating in GE were conducted in the Aeroacoustic facility at the University of Bristol. The anechoic chamber has dimensions of 7.9 m in length, 5.0 m in width and 4.6 m in height, including the surrounding acoustic walls ^46^. An APC propeller with a 10-inch (254 mm) diameter and 5.5 inch pitch angle in a pusher configuration was used. The propeller was mounted at an adequate height, with its rotational axis positioned 1.2 m above the perforated mesh platform of the anechoic chamber to prevent interference with the propeller’s airflow. The geometric profile of the propeller is plotted in the Supplementary Fig. S1. The propeller was driven by a 40 A brushless DC T-motor with 24 poles to reduce the influence of motor noise ^47^. The motor was controlled using a custom-made PID controller via LabVIEW. The control was achieved using a digital pulse width modulated signal with RPM feedback for the PID controller achieved using a PCB electronics LaserTach LT2-ICP tachometer. The RPM control through LabVIEW was achieved with an accuracy of $\pm 5$ RPM.

**Fig. 2:**
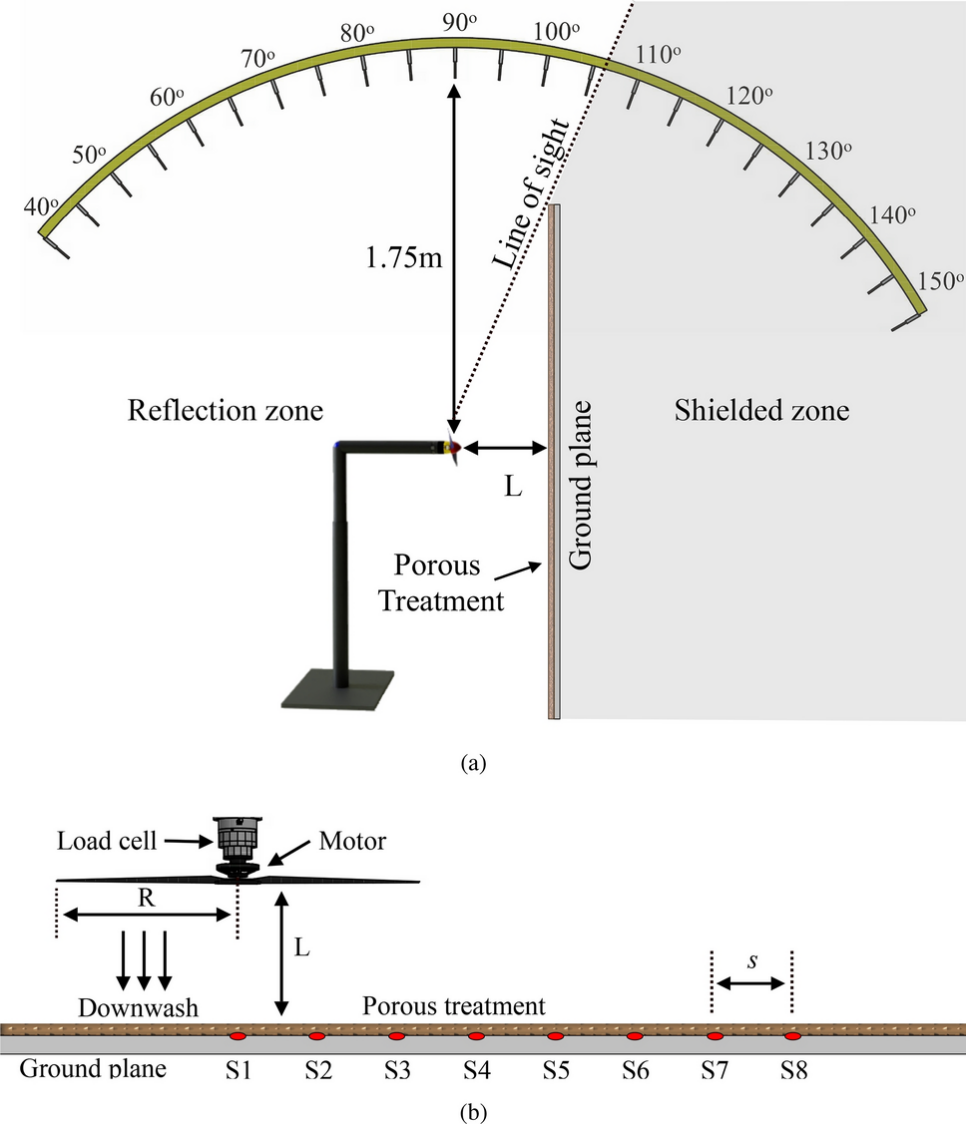
Illustration of the experimental configuration for Ground Effect (GE) investigation, showcasing (a) a lateral perspective with different ground plane (GP) locations highlighting the far-field microphone array, and (b) a detailed view focusing on the propeller (radius; $R = 127~mm$), motor, load cell, and GP instrumentation featuring surface microphones (S1 to S8) positioned $s = 50~mm$ apart, along with key experimental parameters.

In order to study propellers in GE, a rigid flat plate acting as the ground plane (GP) was placed at 8 different positions (L) away from the propeller. At each location, the propeller was set to operate at a constant rotational rate. The flat plate had a length and width of $1.7 m \times 1.5 m$, manufactured using 12 *mm* thick medium-density fibreboard material. The plate surface was instrumented with a G.R.A.S 48LX-8 UTP Line-Array medium pressure microphone which features 8 channels located in the centre of the plate directly below the propeller hub. The 8 flush-mount surface microphones (S1 to S8) were arranged radially, each separated by a distance of $s=50~mm$, beginning with S1 directly below the propeller hub. As shown in Fig. 2, the tests were carried out to induce varying degrees of GE corresponding to $L/R=0.75,1,1.5,2,2.5,3,3.5,$ and 4. The position of the ground plane was adjusted by axially traversing the flat plate away from the propeller plane of rotation. To study the potential of porous materials on the attenuation of noise generated by propellers in GE, three different types of porous foams were applied to the ground plane. These foams, identified by their pore density-45PPI, 75PPI, and 75PPI-T (where T denotes thick)-differ in thickness. The 45PPI and 75PPI foams each have a thickness of 12 mm, while the 75PPI-T foam is thicker, with a 24 mm thickness. Porous materials are fundamentally characterized by their volume porosity $(\phi (\%))$, and airflow permeability $(\kappa )$. The 45 PPI and 75 PPI foams used in this study exhibit porosity of $87.9\%$ and $74.7\%$, respectively. The corresponding airflow permeability are $3.93 \times 10^{-6} \, \text {m}^2$ for 45 PPI foam and $7.7 \times 10^{-9} \, \text {m}^2$ for 75 PPI foam. It is important to note, that since the surface treatments are flow permeable, the plate distances from the propeller are always considered with reference to the Solid plate despite the porous treatments having their own associated thickness.

Far-field noise measurements were acquired using a polar array of 23 microphones distributed in the axial direction centred on the propeller at a distance of 1.75 m ($\approx 13R$). The polar array covers observer locations between $\theta =40^\circ$ above the plate and $\theta =150^\circ$ below the plate with the $\theta =90^\circ$ microphone positioned in the propeller plane of rotation. Measurements were captured within the shielded zone to characterise the noise behaviour of propellers in take-off/landing over high-rise buildings. Measurements were acquired using a 1/4-inch G.R.A.S 40PL microphone with a corrected flat frequency response at frequencies from 10 Hz to 20 kHz, and a dynamic range of 150 dB. Simultaneous measurements were collected with the G.R.A.S 48LX-8 UTP surface pressure microphone with a flat frequency response of up to 100 kHz. The data were captured using a National Instrument PXIe-4499 for $t=16$ s at a sampling frequency of $f = 2^{16}$ Hz. The aerodynamic measurements including thrust and torque were measured using a 60N ATI Mini40E six-axis force and torque sensor. The load data were sampled at $2^{13}$ Hz for 16 s.

The power spectrum results were obtained using the power spectral density (PSD) of the pressure signals with a Hanning window, and the acquired data were averaged 220 times to yield a frequency resolution of $\Delta f=2$ Hz. The sound pressure level (SPL) spectrum can then be calculated using the following equation:

1 $$\begin{aligned} SPL = 10 \cdot \log _{10} \left( \frac{PSD(f) \Delta f}{p_{ref}^2} \right) , \end{aligned}$$

where $p_{\text {ref}}=20~\mu$Pa is the reference pressure. Further, thrust-scaled SPL (TSSPL) values are analyzed for microphones to enable a fair comparison of the noise signature across different test configurations. Thrust scaling is performed using the following equation^48,49^:

2 $$\begin{aligned} TSSPL = 10 \cdot \log _{10} \left( \frac{PSD(f) \Delta f}{p_{ref}^2} \left( \frac{D^2}{T} \right) ^2 \right) , \end{aligned}$$

where, *T* and *D* are the propeller thrust and diameter, respectively. This formulation normalizes the *SPL* facilitating direct comparison of acoustic output per unit thrust across different operational configurations.

## Results and discussion

### Aerodynamic measurements

In order to accurately assess and comprehend the aerodynamic performance of propellers in GE, it is crucial to conduct tests at various distances from the ground. Therefore, the aerodynamic characteristics of the propeller at various proximities ($L/R=0.75,1,1.5,2,2.5,3,3.5$ and 4) to the ground are investigated. The results are presented for Solid ground and three different types of porous surface treatments, along with a benchmark case of the isolated propeller for comparison.

Typically, time-averaged, non-dimensional aerodynamic coefficients are widely used in propeller studies, with the coefficients of thrust ($C_T$) and power ($C_P$) being particularly important. The coefficients are computed using the following equations:

3 $$\begin{aligned} & C_T = \frac{T}{\rho n^2 D^4}, \end{aligned}$$

4 $$\begin{aligned} & P = 2\pi nQ, \end{aligned}$$

5 $$\begin{aligned} & C_P = \frac{P}{\rho n^3 D^5}. \end{aligned}$$

where, $\rho$ represents air density, *n* denotes propeller rotational speed in RPS, *D* signifies propeller diameter, *Q* indicates torque, and *P* refers to power.

**Fig. 3:**
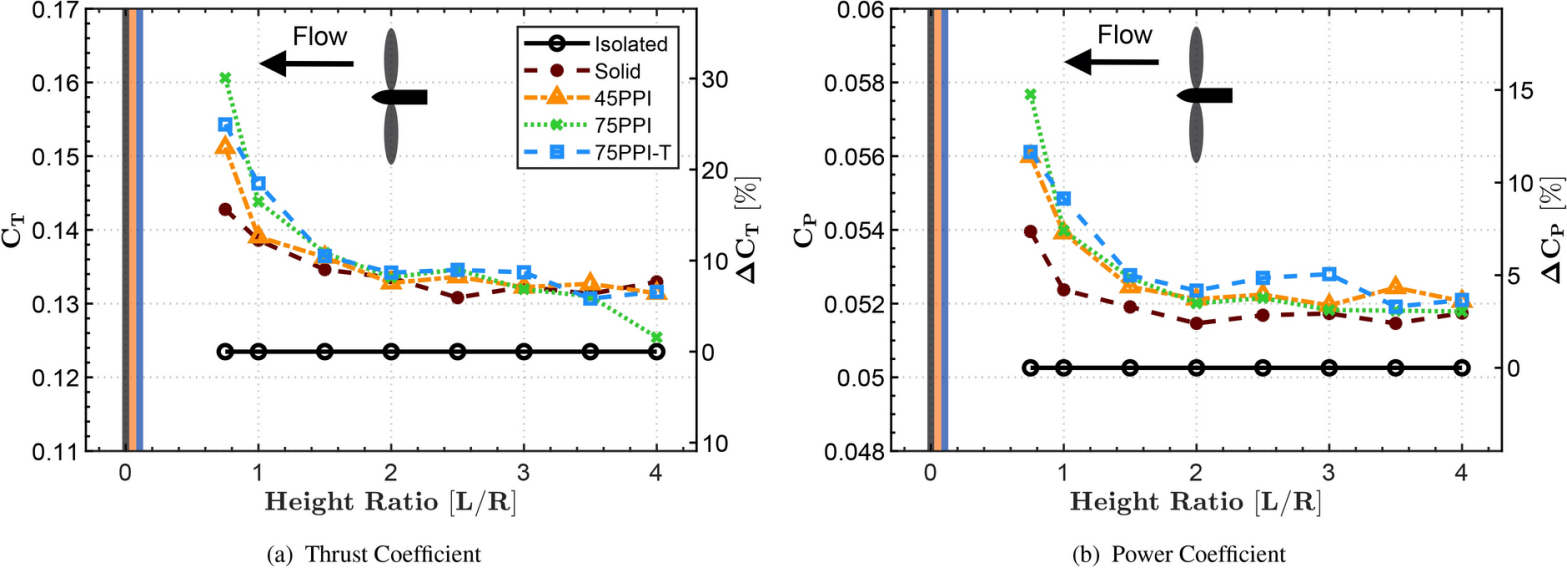
Evolution of aerodynamic coefficients- thrust ($C_T$) and power ($C_P$), with ground distance (*L*/*R*); subjected to 7000 RPM.

The evolution of $C_T$ and $C_P$ is presented in Fig. 3 for parameter = 7000 RPM. The results in this section are presented in terms of increasing plate distance on the x-axis, corresponding aerodynamic coefficients on the left y-axis and relative increments compared to the ’Isolated’ cases on the right y-axis. The three coloured lines at $L/R=0$ are representative of the thickness of the Solid plate and the additional thicknesses of the porous materials. Both 45PPI and 75PPI have the same thickness (12 mm) whilst 75PPI-T is twice the thickness. The results are presented in terms of increasing plate distances for isolated, Solid and porous configurations. For the Solid case, the $C_{T}$ and $C_{P}$ increase at closer plate distances, which is due to the well-known high-pressure cushioning effect that is produced by the presence of a Solid surface in the wake region ^29,38,50,51^. These effects are due to the altered velocity of the rotor slipstream that occurs in close proximity to the ground. Moreover, vertical compression of the wake, increased turbulence and re-circulation are also known to play a role in the aerodynamic alterations. The results in Fig. 3 show that the $C_{T}$ and $C_{P}$ begin to increase exponentially as the propeller enters IGE ($L/R \le 2$). These observations are in line with existing literature ^41^ which found the thrust ratio to increase exponentially within GE at $L/R\le 2$. The $C_{T}$ and $C_{P}$ results for the porous cases show higher levels of thrust and power compared to that of the Solid case. The porous configuration with 45PPI is the coarsest amongst all the tested cases and exhibits values close to the Solid case, with an increase of nearly $20\%$ and $11\%$ in $C_{T}$ and $C_{P}$, respectively, compared to the isolated case. Most interestingly, the porous configurations show increased levels of thrust and torque, with 75PPI recording as much as $30\%$ and $15\%$ increments, respectively, compared to the Solid baseline.

It can be hypothesized that porous surface treatments restrict the radial outflow on the plate after impingement. This restriction partially reflects the propeller wake, enhancing the cushioning effects and leading to increased lift. In practical applications, the augmented ground effect (GE) thrust is projected to enhance aircraft efficiency at lower RPMs. By strategically managing torque, the engine load can be reduced, optimizing performance. This fine-tuning minimizes fuel consumption and mechanical stress on the engine, particularly during takeoff and landing phases. Consequently, aircraft efficiency improves, and engine lifespan extends as a result of less strenuous operation conditions.

### Far-field measurements

Far-field acoustic behaviour is crucial for comprehending the operational impacts of propellers, particularly within UAM applications. This section delves into the detailed acoustic characterization of propellers, analysing their complex noise spectrum and providing essential insights for targeted noise mitigation and aerodynamic optimization in UAM operations. The subsequent analysis focuses on the tonal and broadband noise, influenced by a variety of operational and environmental factors.

Tonal noise, predominantly originating from blade pass frequency (BPF) and its harmonics, emanates mainly from propeller loading and, thickness noise (negligible in subcritical tip Mach number conditions ($< 0.7$) in the current study)^52^. Blade-vortex interaction (BVI), especially prevalent in hover, significantly contributes to tonal noise via unsteady pressure fluctuations. The “Haystacking” phenomenon intensifies the acoustic behaviour, inducing broad, quasi-periodic spectral peaks at the BPF and its harmonics due to prolonged turbulent eddies correlating across blades^52^. Additionally, broadband noise, characterized by its stochastic nature, predominantly emerges from turbulent interactions over the propeller, integrating both trailing-edge noise from the turbulent boundary layer and leading-edge turbulence ingestion.

In GE conditions, the interaction between the blade wake and the propeller becomes more pronounced, significantly enhancing tonal noise due to the propeller’s proximity to the ground plane. This proximity leads to increased flow recirculation, where the airflow near the ground is disturbed and reflects back towards the propeller, creating complex flow interactions that contribute to higher noise levels. Additionally, ground effect amplifies the blade-vortex interaction (BVI), where the rotor blades interact more intensely with the vortices generated by previous blades, further increasing both tonal and broadband noise. Hover conditions amplify these GE-induced effects, leading to stronger acoustic emissions and a noisier flow field.

The experimental study involves far-field noise measurements for two distinct propeller configurations, i.e. Isolated, and in GE. Employing a vertically oriented Solid plate as a GP, this research investigates the impact of GE on propeller noise. A non-dimensional BPF number ($m = f$/BPF) with $m = 1$ equivalent to 233.33*Hz* is formulated to simplify and facilitate in-depth acoustic analyses. This methodological framework is designed to extract key insights into the acoustic behaviour of propellers under various operational conditions.

#### Overall sound pressure level

In the context of drone operations, particularly during takeoff or landing, evaluating the directivity of generated noise is important. A thorough understanding of noise directivity facilitates possible improvements in vertiport and aircraft designs, enhancing societal acceptance and compliance with noise regulations. The A-weighted OASPL is calculated by applying a filter to the PSD before integrating over the frequency range of $150-20000$ Hz using a reference pressure of $p_{ref}=20~\mu$Pa, as expressed below:

6 $$\begin{aligned} OASPL~(dBA) = 10 \cdot \log _{10} \left( \frac{\int PSD(f) \cdot W_A(f) \, df}{p_{\text {ref}}^2} \right) . \end{aligned}$$

where, $W_{A}(f)$ is the A-weighting factor applied at each frequency *f*. Using A-weighting enables the analysis to more accurately reflect the perceived loudness of drone noise by human listeners and provides a metric that is closely aligned with community noise standards.

**Fig. 4:**
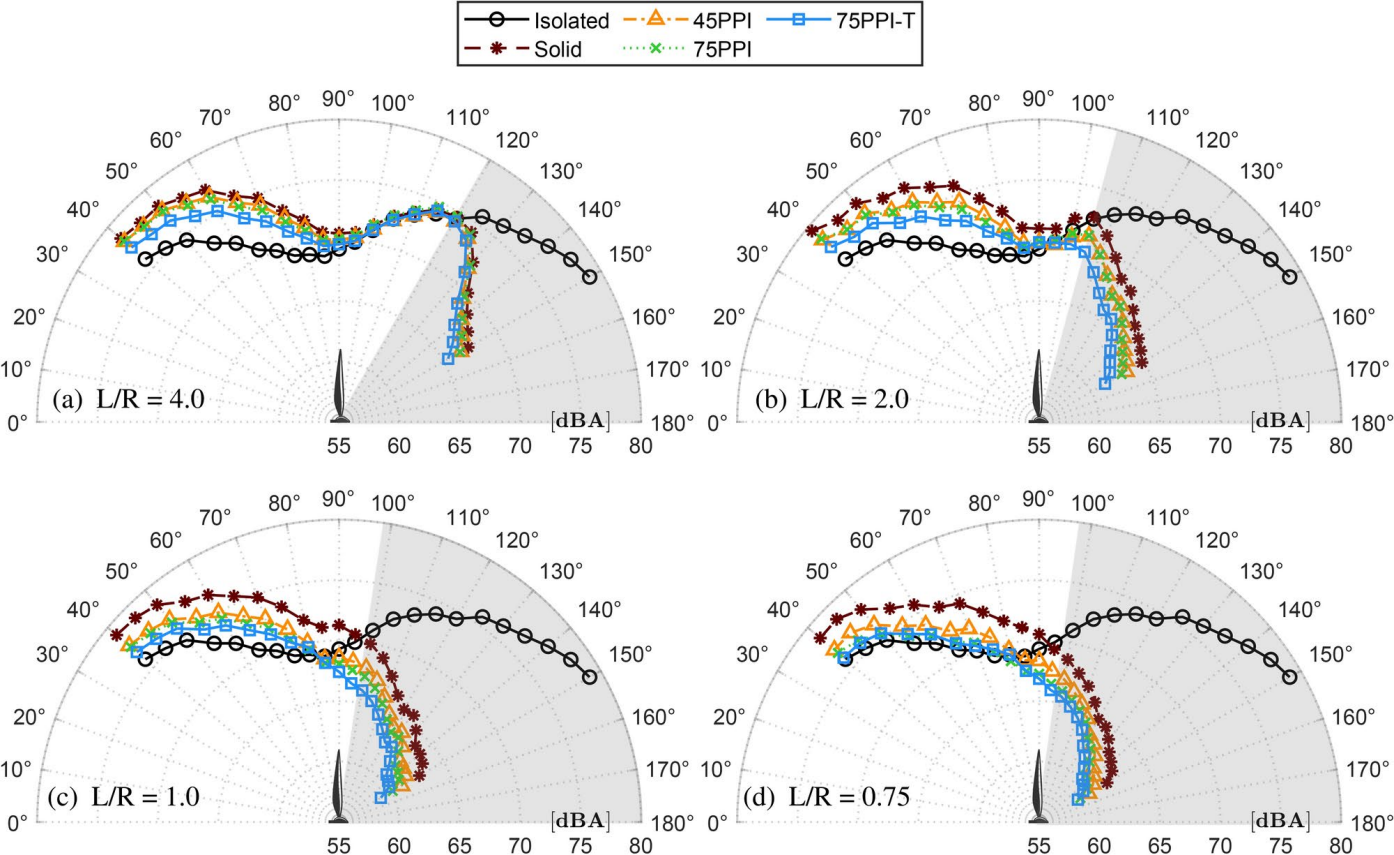
Directivity pattern of A-weighted OASPL for propeller operating at 7000 RPM, evaluated at multiple ground distances (*L*/*R*).

The polar directivity plots of the OASPL for the test plate distances for both IGE and OGE conditions are shown in Fig. 4. The shaded region in each plot indicates the shielded region below the GP. The OASPL results for the Isolated case (without GP) at lower angles ($\theta < 90^\circ$), show a gradual decrease, consistent with the dominance of thickness noise, which is more significant along the axis of rotation^53^. This decrease continues as the angle increases, reflecting the reduced contribution of loading noise in the sideline region^54^. However, beyond $\theta > 100^\circ$, the OASPL for the isolated case exhibits a noticeable increase, peaking around $\theta = 150^\circ$. This rise in noise levels is primarily attributed to the wake interaction and loading noise effects in the downstream region, where the turbulent wake interacts with the ambient air, leading to enhanced acoustic emissions^53^. In contrast, introducing a ground plane modifies this behavior. At lower angles ($\theta < 90^\circ$), the OASPL remains elevated due to ground reflections amplifying the thickness and loading noise. As the angle increases, the noise reduction is less pronounced compared to the isolated case, since the GP continues to reflect and sustain higher noise levels. Notably, beyond $\theta > 100^\circ$, the OASPL sharply decreases in the GP case as the microphones are shielded by the ground plane.

The directivity plots in Fig. 4(a) for OGE condition at $L/R = 4.0$ shows minimal ground effect, with OASPL trends closely following the isolated case. Porous ground plates offer limited noise attenuation, especially at lower polar angles. As ground proximity decreases to $L/R = 2.0$ in Fig. 4(b), OASPL begins to increase at shallower angles ($\theta < 90^\circ$) due to ground reflection effects. Porous treatments start to show their benefits by reducing noise at these angles. At closer distances corresponding to IGE conditions ($L/R \le 1.0$), the ground effect becomes more pronounced. The solid ground plate amplifies OASPL by $\approx$3 dBA at shallow angles, while porous treatments reduce this increment by $\approx$2 dBA, demonstrating their effectiveness at IGE $L/R = 1.0$ shown in Fig. 4(c). The ground effect is strongest at $L/R = 0.75$ in extreme IGE conditions, as shown in Fig. 4(d). Here, the thickest porous configuration achieves the most significant noise reduction caused by the GP. This reduction in ground effect, and the subsequent noise, underscores the efficacy of porous treatments in mitigating adverse flow interactions, particularly in IGE conditions.

#### Sound pressure level

The noise generation mechanisms of the propeller were investigated under various GE conditions, with a focus on the effects of porous surfaces on the radiated noise. Readers are encouraged to refer to the supplementary data through Supplementary Fig. S2-S5 for comprehensive far-field spectral examination. This investigation enhances the broader understanding of noise control strategies during take-off and landing for UAM, emphasizing the importance of ground treatments with specific surface textures and porosity in improving aerodynamic and aeroacoustic performance.

**Fig. 5:**
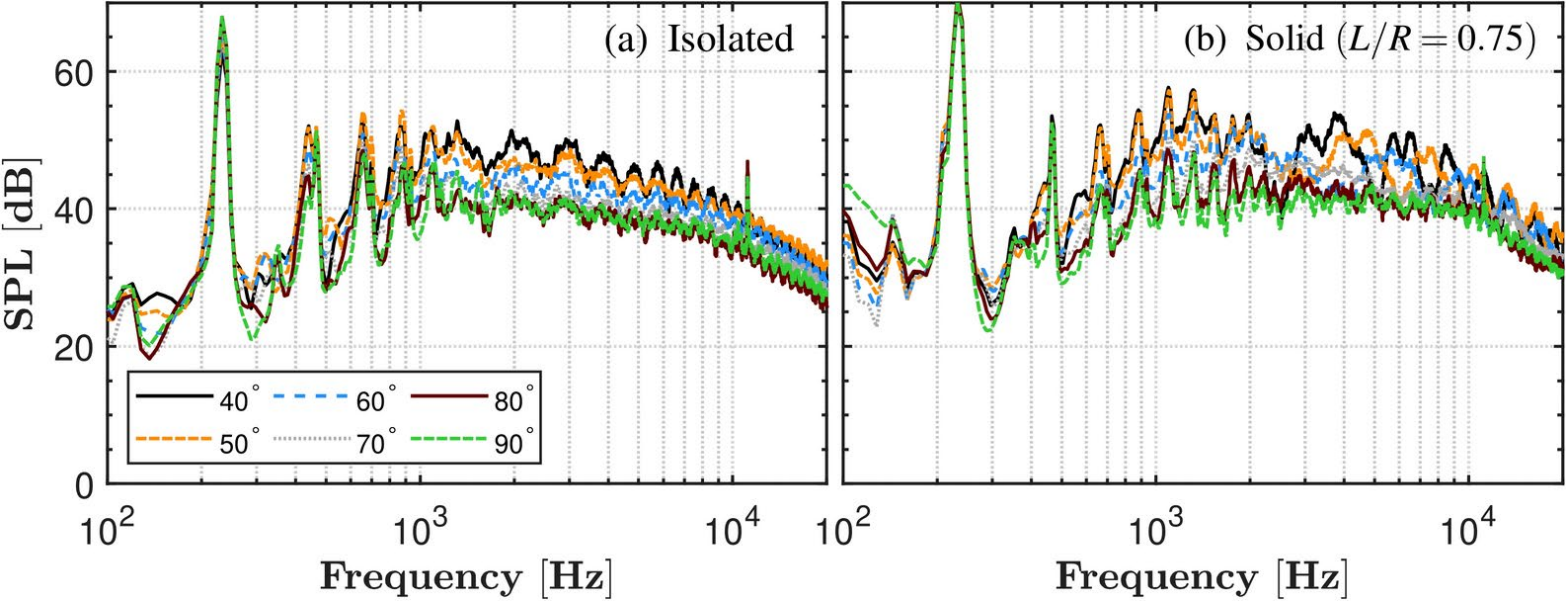
Far-field SPL of the propeller operating at 7000 RPM under Isolated and GE conditions.

The SPL spectra in Fig. 5 compare the Isolated and Solid plate configurations ($L/R=0.75$) across directivity angles from $40^\circ$ to $90^\circ$, analyzed over a frequency range of 100 - 20,000 Hz. The SPL spectra revealed clear distinctions between the Isolated and Solid plate configurations. The SPL predominantly exhibited discrete tones at the BPF and its harmonics, along with a range of mid- to high-frequency noise. For the BPF and the first harmonic, the Solid plate case displayed a relatively higher SPL compared to the Isolated case, especially at smaller observer angles ($\theta =$ $50^\circ$). The Solid plate configuration further revealed additional mid-frequency harmonics ($600-2500$ Hz, $~m = 6 - 9$), absent in the Isolated case. This is often attributed to unsteady loading acting on the blades, caused by either incoming flow turbulence, or the turbulence caused by the propeller. In the case of a propeller operating near a solid plate, additional peaks in the SPL spectra are observed. These emerge due to complex aerodynamic interactions. The proximity to the GP leads to turbulent ingestion as the propeller’s wake is reflected and recirculated by the GP, which can generate higher-order harmonics such as blade-vortex interaction (BVI). Furthermore, the ’air-cushion’ effect induced by GP causes alterations in the wake flow, amplifying turbulence and potentially leading to greater noise emissions and more pronounced spectral peaks^29^. The Solid plate case demonstrated more complex directivity patterns, with a significant increase in higher harmonics observed at relatively shallower angles compared to the sideline. Notably, distinct broadband peaks appeared in the high-frequency range from ($3.8< f < 13$ kHz), suggesting that turbulence ingestion into the blade significantly influences acoustic behaviour. A singular high-frequency peak ($f > 10$ kHz) observed in the SPL spectra is attributed to mechanical noise originating from the motor.

**Fig. 6:**
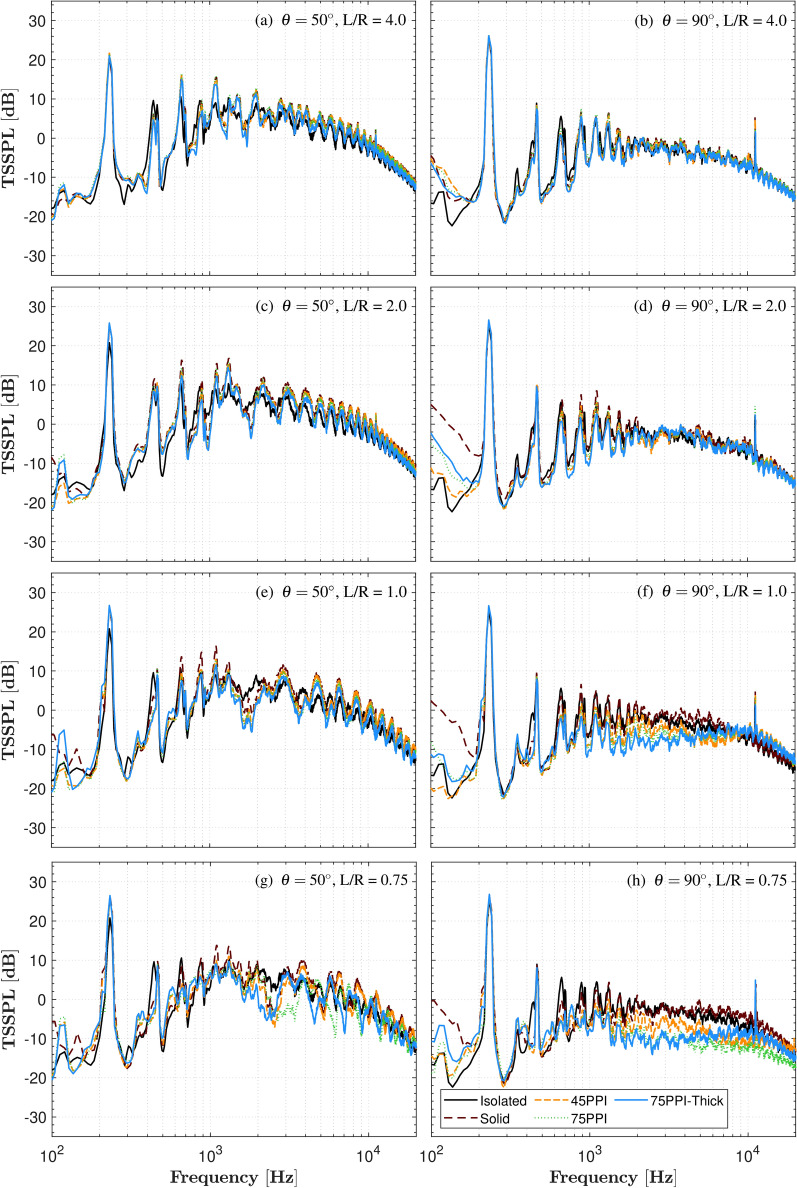
Thrust-scaled far-field noise spectra (TSSPL) of propeller operating at 7000 RPM, recorded at $\theta =50^\circ$ and $90^\circ$, subjected to various ground distances (*L*/*R*).

The TSSPL plots in Fig. 6 compare Isolated and Solid plate configurations at directivity angles $\theta = 50^\circ$ and $\theta = 90^\circ$, selected to capture key acoustic features in the reflected ($\theta =50^\circ$) and sideline ($\theta =90^\circ$) regions. These angles are essential for evaluating noise exposure outside shielded zones in urban environments. Our study extends beyond ground-level scenarios to inform shielding and reflection effects for UAM vehicles operating near varying building heights, where noise may propagate from rooftops to nearby taller structures. The noise spectra reveal distinct differences between the Isolated and Solid plate configurations, with pronounced discrete tones observed at the BPF and its harmonics, accompanied by a spectrum of mid- to high-frequency noise.

The OGE results at $L/R=4$ are presented in Figs. 6a and 6b for $\theta =50^\circ$ and $\theta =90^\circ$, respectively. At $\theta =50^\circ$, the GE configuration exhibits a significant increase in TSSPL by approximately 4.0-8.0 dB for frequencies above 1000 Hz. This increase is likely due to the reflection of noise from the ground plane, which is observed in both solid and porous configurations. As expected, this increase is absent at $\theta =90^\circ$, where the sideline region does not experience reflections from the ground plane. A similar trend is also observed at the OGE region at $L/R=2$.

In the IGE region at $L/R=1$, the BPF tone is more pronounced for the GP configurations, which aligns with the thrust benefits observed in load measurements. This correlation is expected, as increased thrust typically results in a stronger BPF tone. At $\theta =50^\circ$ (Fig. 6e), The solid configuration also shows elevated tonal peaks at the harmonics ($f=400-2000$ Hz) compared to the isolated condition, with the porous configuration slightly reducing these peaks. Interestingly, despite the porous configuration exhibiting the highest BPF, it produces relatively lower harmonic peaks. In the mid- to high-frequency range ($f>2000$ Hz), both solid and porous configurations display broadband humps not seen in the isolated case. These humps could be attributed to the enhanced turbulence and shear layer interactions caused by the ground plane, which are further influenced by the porous material’s effect on the flow field. At $\theta =90^\circ$ (Fig. 6f), which represents the sideline position, the solid configuration exhibits an increase in noise levels within the mid- to high-frequency range. In contrast, the porous configurations, particularly the 75PPI and 75PPI-T treatments, show a noticeable reduction in noise within this frequency range. This difference can be attributed to the higher wall jet flow velocity in the solid configuration, which enhances noise generation in the sideline region. Conversely, the porous configurations reduce this outward flow velocity, likely due to the increased surface roughness introduced by the porous materials. This roughness disrupts the flow, diminishing the velocity and thereby reducing the noise. Additionally, it is postulated that the radial out-wash may be tripped and entrapped by the pores, leading to increased boundary-layer turbulence that is subsequently mitigated within the porous structure. Furthermore, the porous treatments may contribute to the formation of an air cushion beneath the propeller, further aiding in noise reduction by altering the flow dynamics near the ground plane. This cushioning effect, combined with the flow deceleration, likely plays a crucial role in mitigating noise in the mid- to high-frequency range for the porous configurations in the sideline regions.

At the closer IGE position of $L/R=0.75$, the tonal peaks at the harmonics further decrease for the porous configurations compared to the solid plate, relative to the $L/R=1$ position. However, the broadband humps become more irregular, with distinct variations across different porous treatments. This irregularity is likely due to the enhanced proximity to the ground plane, which intensifies flow interactions, including increased turbulence, flow reflection, and modifications to the wake structure and shear layers. These effects are specific to the IGE configuration and are absent in the isolated propeller case, highlighting the significant influence of ground effect and porous materials on the overall acoustic behaviour. As previously observed for $L/R=1$, the results at $L/R=0.75$ similarly show a significant reduction in noise levels for the porous configurations compared to the solid configuration. At $\theta =90^\circ$ and $L/R=0.75$ (Fig. 6h), a significant TSSPL reduction of approximately 13 dB is observed in the frequency range of 1500-10000 Hz for the porous configuration compared to the solid configuration. Overall, greatest reduction is achieved with the 75PPI-T treatment, while the 45PPI treatment shows the least reduction. These results clearly demonstrate the effectiveness of porous treatments in mitigating noise, highlighting their potential benefits for drone landing pads in reducing acoustic impact during operations.

### Near-field characteristics

**Fig. 7:**
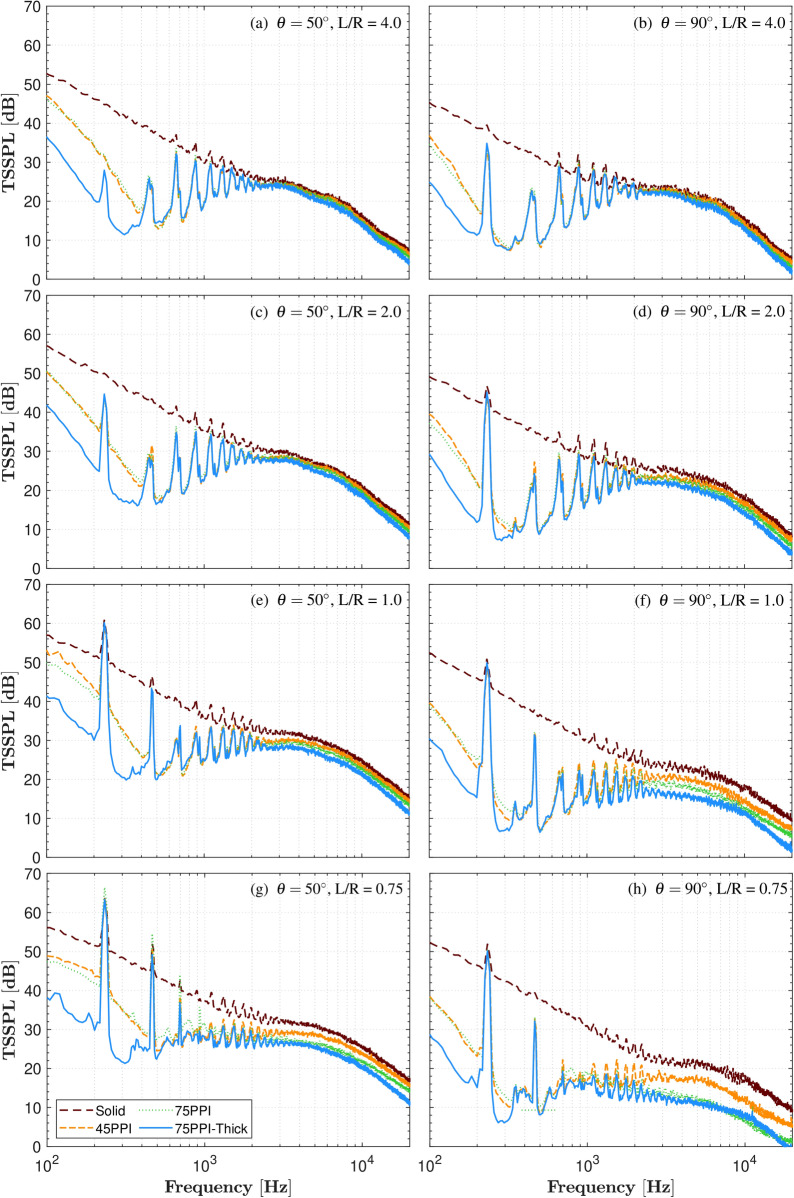
Thrust-scaled near-field noise spectra (TSSPL) of propeller operating at 7000 RPM, recorded at S4, and S8 positions, subjected to various ground distances (L/R).

Following the far-field acoustic assessment, this section transitions to an examination of the propeller’s near-field acoustic behavior under various GE conditions. The near-field spectra, from S1 to S8 across different configurations and ground proximities, are analyzed to gain a deeper understanding of the acoustics. Readers are referred to Supplementary Figs. S6-S9 for a comprehensive overview of the results. For conciseness, the discussion here focuses only on the near-field thrust-scaled noise spectra (TSSPL) at S4 and S8, as presented in Fig. 7. The data demonstrate the presence of the BPF and its harmonics, which intensify as the ground distance (*L*/*R*) decreases, with a noticeable reduction at positions farther from the propeller tip (S4 to S8). Particularly at S4, located near the propeller tip, there is a significant amplification of low-frequency broadband noise, compared to the inner microphones (S1-S3), attributed to direct wake impingement (see Figs. 7a, c, e, g). Across all configurations, the porous treatments consistently show substantial noise reduction, especially in the low-to-mid frequency broadband spectra.

In the OGE scenarios ($L/R = 4.0$ and $L/R = 2.0$) shown in Figs. 7a-d, the porous treatments demonstrate significant noise reduction. The 75PPI-T configuration consistently achieves the highest TSSPL reductions, with up to 30 dB in low frequencies and 4.0-5.0 dB in high frequencies at the propeller tip region (S4). At the S8 position, reductions reach up to 30 dB in low frequencies and 5.0-6.0 dB in high frequencies. This enhanced noise reduction is attributed to the thicker porous structure of the 75PPI-T configuration, which enhances the “scrubbing effect” by increasing internal friction and turbulence, thereby more effectively dissipating acoustic energy compared to other porous treatments ^55,56^.

As the propeller operates in IGE conditions ($L/R = 1.0$), the TSSPL naturally increases due to the reduced distance to the GP. In GE ($L < 2R$), the BPF at 200 Hz reaches a peak TSSPL of 66 dB for porous configurations at S4, as shown in Figs. 7 (g). Despite this closer proximity, the porous surfaces outperform the solid plate, effectively mitigating the typical noise amplification associated with GE. The BPF and its harmonics are prominent, with the 75PPI-T configuration achieving greatest TSSPL noise reductions of up to 27-36 dB in the low-to-mid frequency range, while the 75PPI and 45PPI configurations achieve up to 30 dB reductions, compared to solid plate configuration. Additionally, the 75PPI-T treatment provides TSSPL suppression of approximately 5.0-10 dB in the mid-to-high frequency range across the radial positions S4 to S8.

In the extreme IGE scenario ($L/R = 0.75$), the 75PPI-T treatment consistently offers the most effective noise attenuation, significantly reducing the increased noise levels, as shown in Figs. 7 (g and h). A reduction of approximately 26-36 dB in the low-frequency range and 6-10 dB across the mid-to-high frequency range is observed at the S4 and S8 positions, respectively, compared to the solid plate configuration. These results emphasise the efficacy of the 75PPI-T porous treatment in mitigating near-field noise across various ground proximity scenarios, particularly in challenging IGE conditions.

### Coherence analyses

This section explores coherence analysis to understand the spatial consistency and propagation of acoustic signals at different configurations and locations. Coherence analysis quantifies the linear relationship between signals captured by two distinct microphones, thereby identifying shared or interconnected influences emanating from noise sources. The magnitude squared coherence $(\gamma ^2)$ is utilized to compute this relationship, defined as:

7 $$\begin{aligned} \gamma ^2_{P_iP_j}(f) = \frac{|\phi _{P_iP_j}(f)|^2}{\phi _{P_iP_i}(f)\phi _{P_jP_j}(f)}. \end{aligned}$$

here, $\phi _{P_iP_j}$ represents the cross spectral density between pressure signals $P_i$ and $P_j$.

**Fig. 8:**
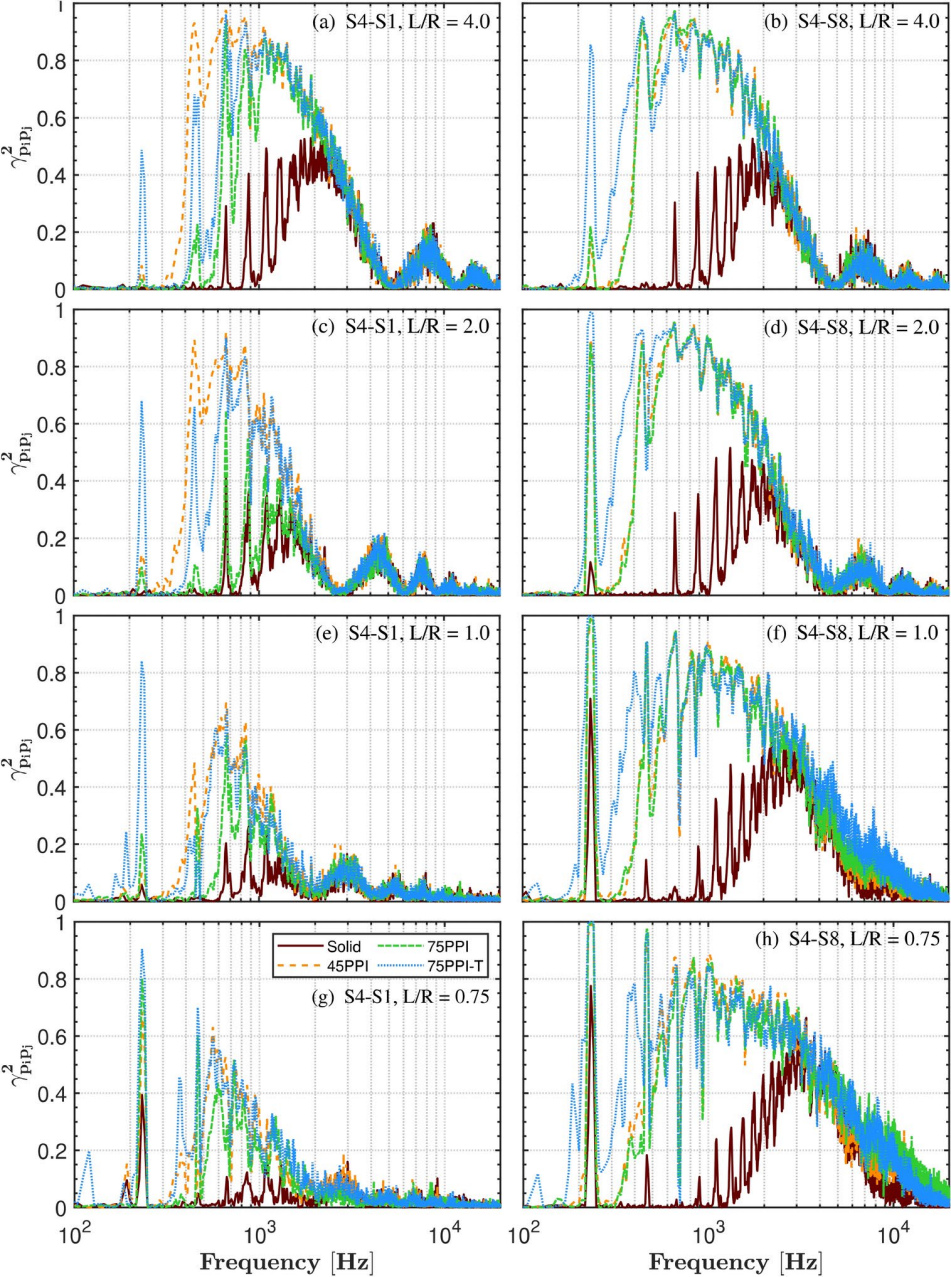
Coherence between pairs of microphones: inner (S1-S4), and outer (S4-S8), for propeller operating at 7000 RPM, subjected to various ground distances (L/R).

Coherence analysis was conducted between the inner (S1-S4) and outer (S4-S8) surface microphones to gain insight into the spatial acoustic characteristics across the propeller span and beyond, as shown in Fig. 8. In general high coherence is observed for the porous treatment. This could be attributed to the porous surface’s ability to absorb and diffuse turbulent energy, reducing random scattering and secondary turbulence. By preserving the organized flow structure, the porous treatment leads to more correlated pressure fluctuations, further enhancing coherence. This is also in line with VanDercreek et al. ^57^ who showed enhanced coherence by suppressing turbulent fluctuations using melamine foam in cavities without impacting the acoustic signals.

Under OGE conditions ($L/R = 4.0$ and $L/R = 2.0$), the coherence between surface microphone pairs is notably high, particularly at the hub, blade tip, and further out positions. This high coherence is likely due to the well-developed turbulent wake of the propeller at these heights, resulting in more organized and correlated unsteady pressure signals. The coherent wake structure and the absence of strong ground interference allow the turbulence to maintain its organization over a larger area, leading to increased coherence between the microphone pairs. At the distance of $L/R = 2.0$, presented in Figs. 8 (c and d), the coherence spectra display a broadband hump that shifts toward higher frequencies compared to $L/R = 4.0$, indicating a consistent spatial and temporal flow across the propeller span. Similarly, the outer microphones (S4-S8) exhibit high coherence, suggesting reduced interference in the flow field farther from the GP.

As the propeller transitions into IGE conditions ($L/R = 1.0$), notable changes are observed in the coherence levels. Compared to the OGE conditions, the coherence diminishes, particularly between the inner microphones (S1-S4). This reduction in coherence is due to the restricted spatial and temporal domains available for flow development as the propeller moves closer to the GP. The decreased distance between the propeller and the GP inhibits the formation of coherent flow structures, resulting in increased turbulence and chaotic flow interactions, which subsequently destabilises the pressure field and reduces overall coherence.

In the extreme IGE scenario ($L/R = 0.75$), these effects become more pronounced. The coherence between both the inner (S1-S4) and outer (S4-S8) microphones shows a significant reduction compared to the OGE cases. The extreme proximity to the GP limits the spatial domain for coherent flow structures to develop, resulting in chaotic and turbulent flow behavior, which further diminishes the coherence. The broadband hump observed in OGE conditions becomes less pronounced, and coherence significantly diminishes at both low and high frequencies.

## Conclusion

This study examines the aerodynamic and aeroacoustic performance of propellers in GE with porous surface treatments, yielding critical insights for UAM. Results indicate that porous materials, particularly 75PPI-T foam, can reduce noise levels by up to 30 dB in low frequencies and 5-10 dB in mid-to-high frequencies compared to a solid ground surface. This noise reduction is most pronounced at lower observer angles, where acoustic impacts are typically greatest, highlighting the potential of porous surfaces to lower propeller noise and enhance compliance with regulatory standards for UAM operations.

Near-field acoustic analysis reveals significant noise reduction along the propeller span under varying ground proximities and porous ground surface treatments. Specifically, porous surfaces in IGE conditions achieve notable suppression, reducing low-frequency noise by 26-36 dB and mid-frequency noise by 6-10 dB with 75PPI-T treatments. These results highlight the complex interactions between propeller wake flow and the ground plane, offering valuable guidance for optimizing surface treatments to reduce UAM noise. Additionally, coherence analysis reveals that porous treatments enhance near- to far-field coherence in the low- to mid-frequency range, improving spatial consistency and predictability. This improvement is crucial for developing effective noise control strategies and understanding sound propagation in complex urban settings.

The study offers key insights for UAM noise reduction, supporting quieter, community-friendly operations and expanding route options in noise-sensitive areas through optimized porous ground treatments. Exploring new materials, such as bio-inspired porous structures or natural materials like grass and mosses, could further enhance vertiport designs. This approach will support the development of efficient, sustainable, and community-friendly urban air transportation.

## Supplementary Information

Supplementary Information.

## Data Availability

The datasets generated during and/or analyzed during the current study are available from the corresponding author on reasonable request. Supplementary data supporting the findings of this study, including various plots and additional analyzes, are available in the supplementary PDF file accompanying this manuscript.
