# Supplementary material for: Porous ground treatments for propeller noise reduction in ground effect
Source: Sci Rep. 2025 Jan 22;15:2170. doi: 10.1038/s41598-024-82876-9 (PMC11754600; doi:10.1038/s41598-024-82876-9)
Supplement: Supplementary file 1 — Supplementary Information. [file 41598_2024_82876_MOESM1_ESM.pdf]

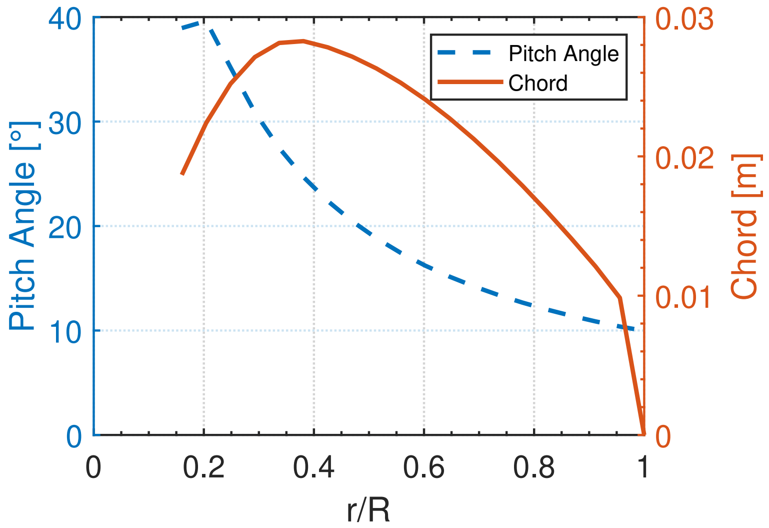

Figure S1. Geometric profile of the APC 10" x 5.5" propeller used in the experimental campaign.

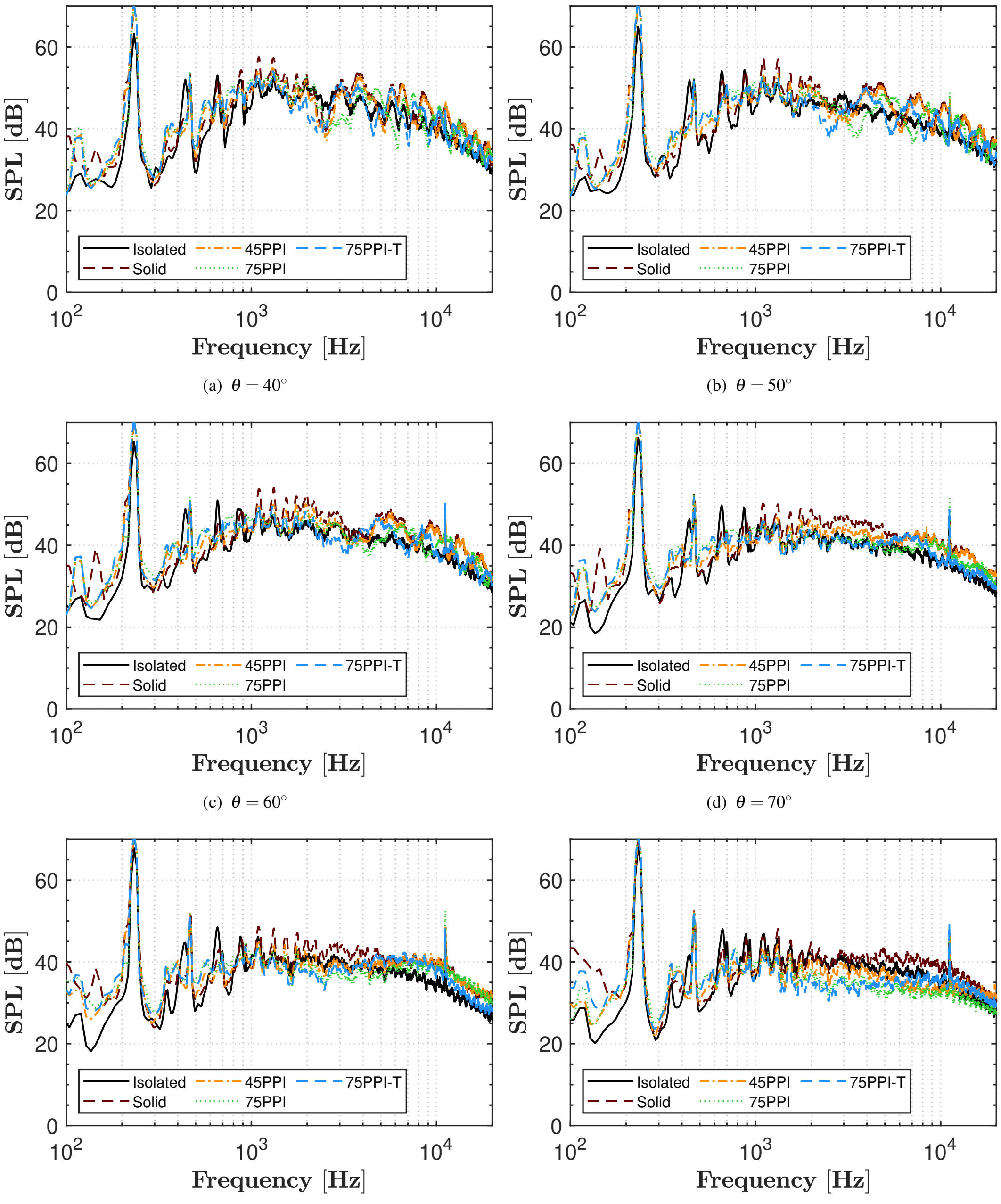

(a) $\theta = 40^\circ$ (b) $\theta = 50^\circ$

(c) $\theta = 60^\circ$ (d) $\theta = 70^\circ$

(e) $\theta = 80^\circ$ (f) $\theta = 90^\circ$

Figure S2. Far-field SPL of propeller operating at 7000 RPM, recorded over directivity angles of $\theta = 40^\circ - 90^\circ$ subjected to ground proximity of $L/R = 0.75$.

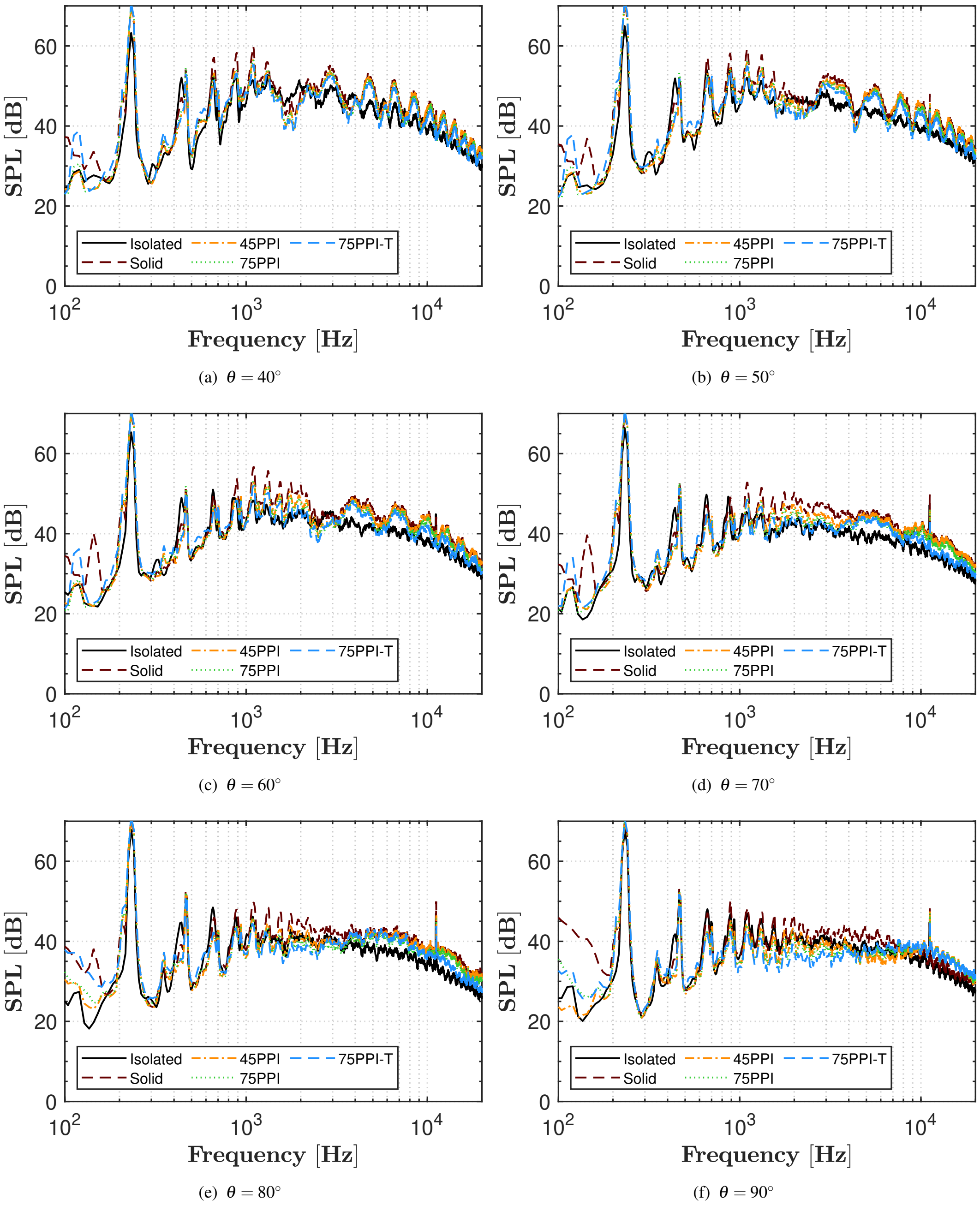

Figure S3. Far-field SPL of propeller operating at 7000 RPM, recorded over directivity angles of $\theta = 40^\circ - 90^\circ$ subjected to ground proximity of $L/R = 1.0$.

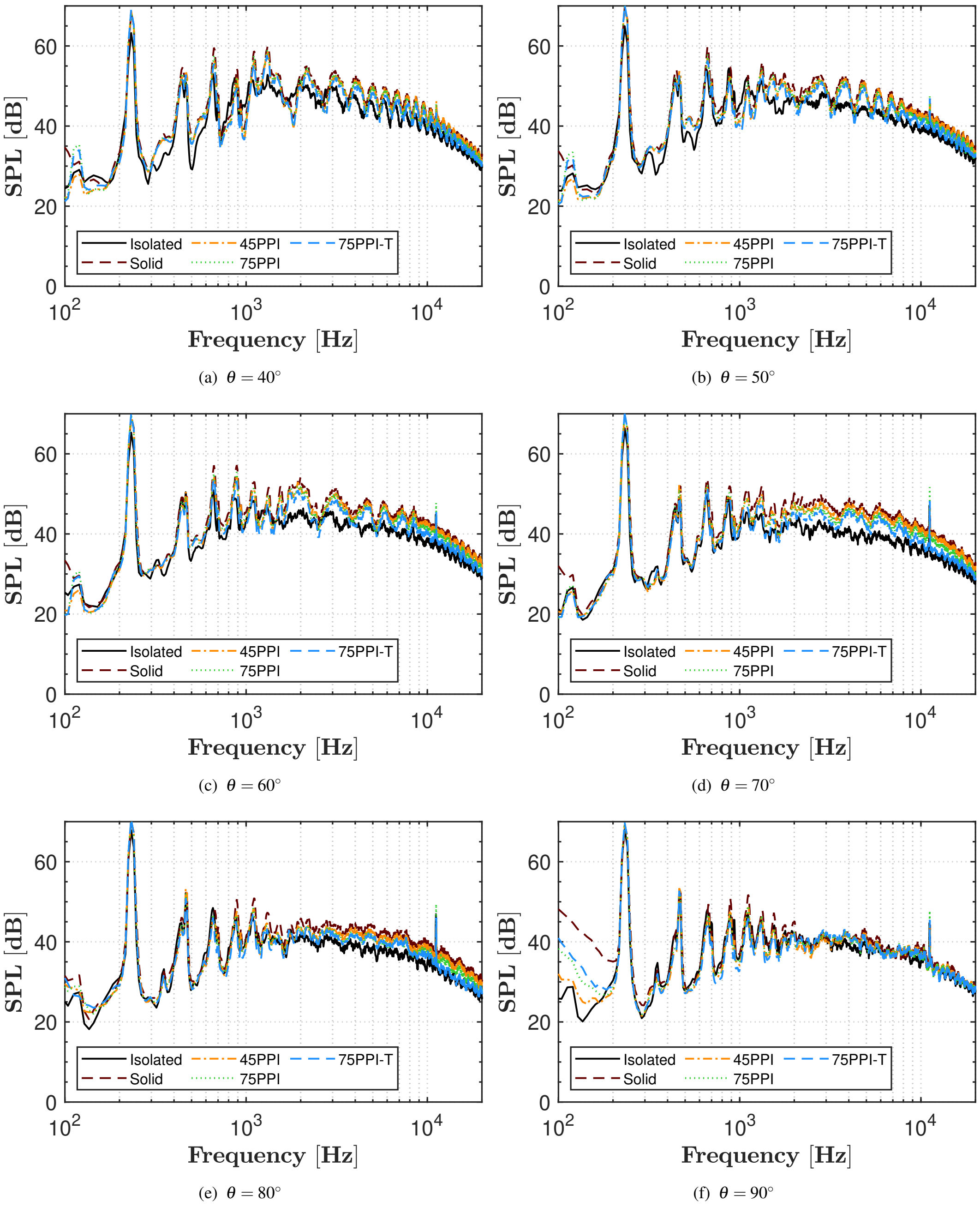

Figure S4. Far-field SPL of propeller operating at 7000 RPM, recorded over directivity angles of $\theta = 40^\circ - 90^\circ$ subjected to ground proximity of $L/R = 2.0$.

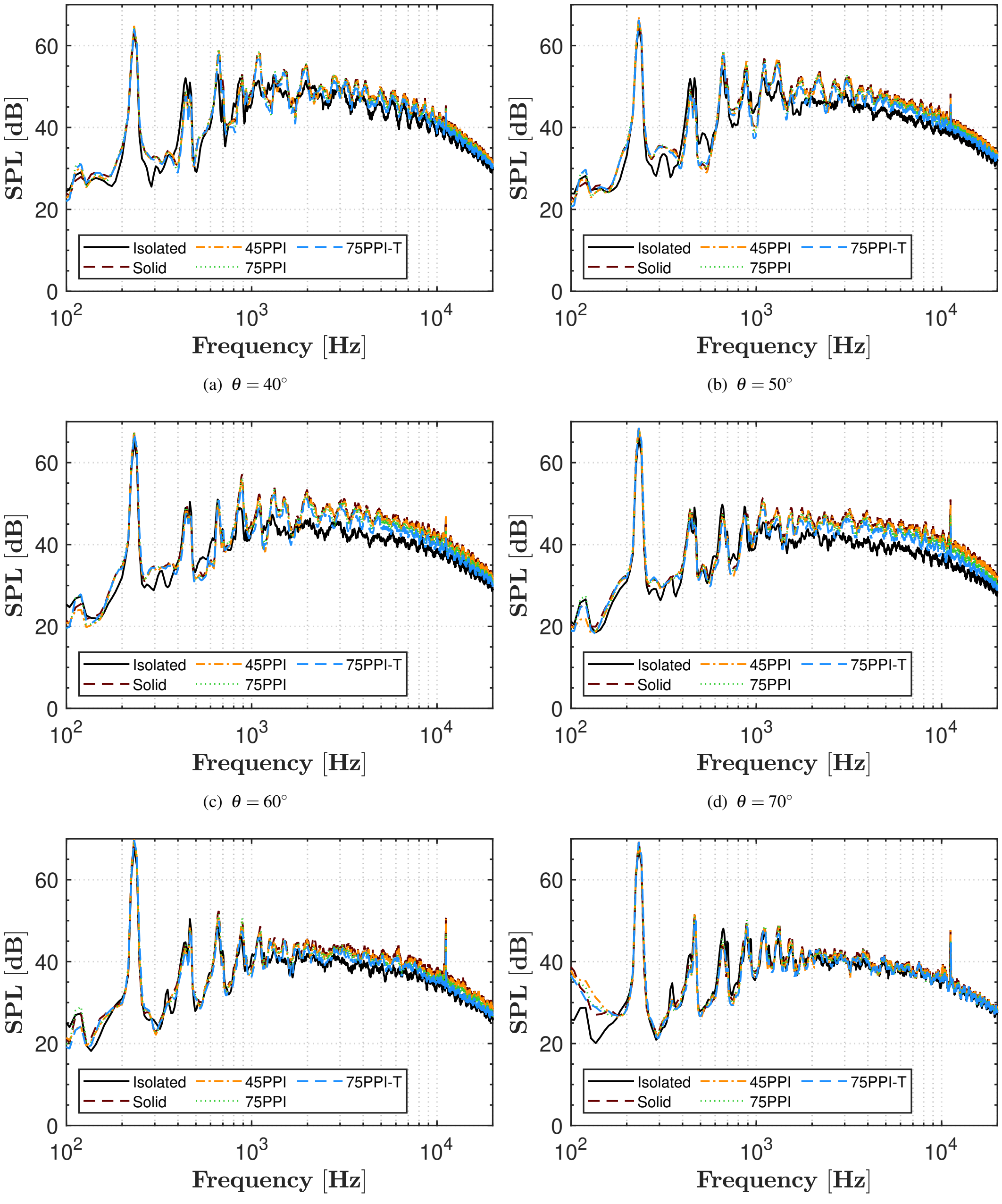

(a) $\theta = 40^\circ$ (b) $\theta = 50^\circ$

(c) $\theta = 60^\circ$ (d) $\theta = 70^\circ$

(e) $\theta = 80^\circ$ (f) $\theta = 90^\circ$

Figure S5. Far-field SPL of propeller operating at 7000 RPM, recorded over directivity angles of $\theta = 40^\circ - 90^\circ$ subjected to ground proximity of $L/R = 4.0$.

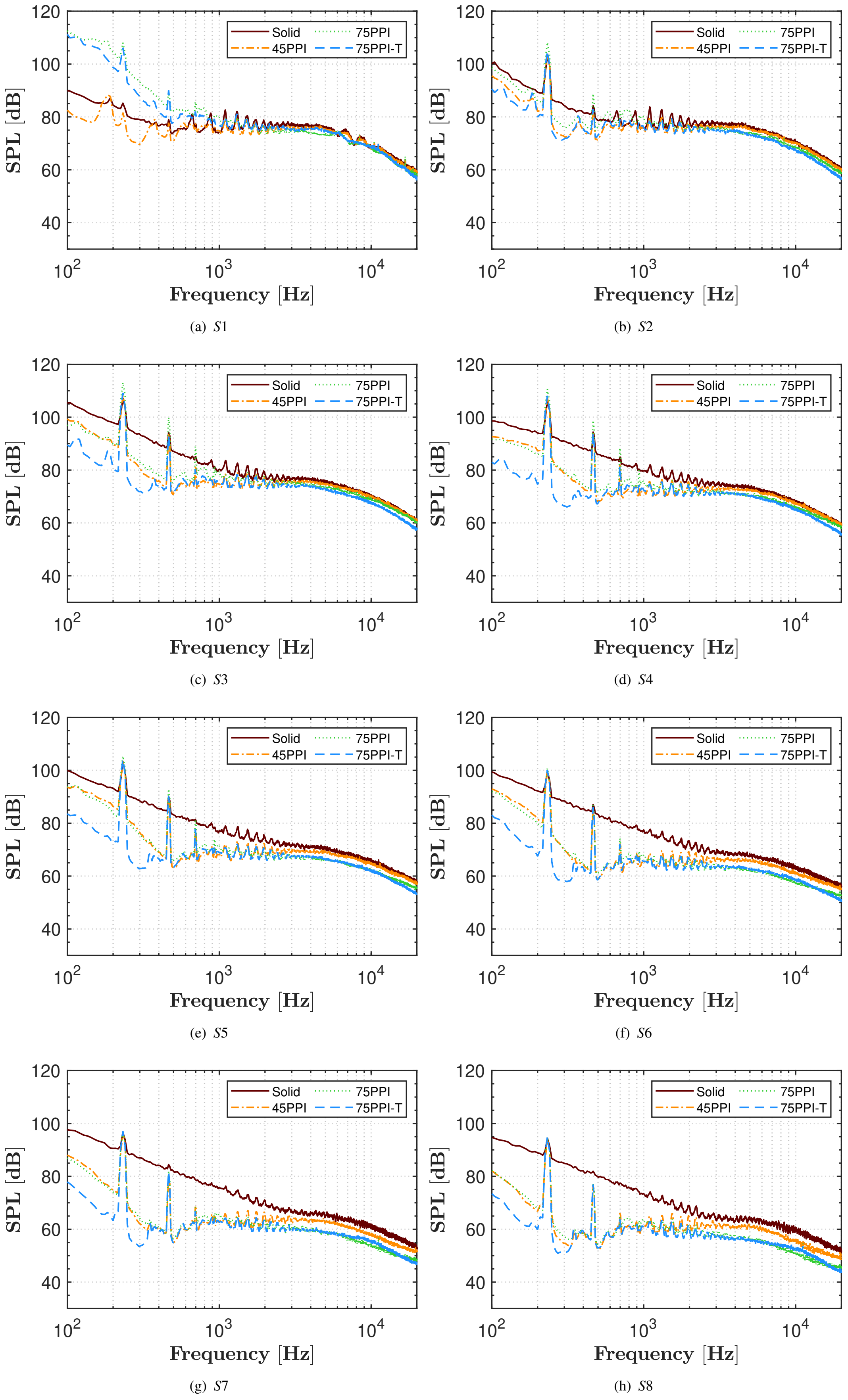

(a) $S1$ (b) $S2$ (c) $S3$ (d) $S4$ (e) $S5$ (f) $S6$ (g) $S7$ (h) $S8$

Figure S6. Near-field SPL of propeller operating at 7000 RPM, across $S1-S8$ microphone positions subjected to ground proximity of $L/R = 0.75$.

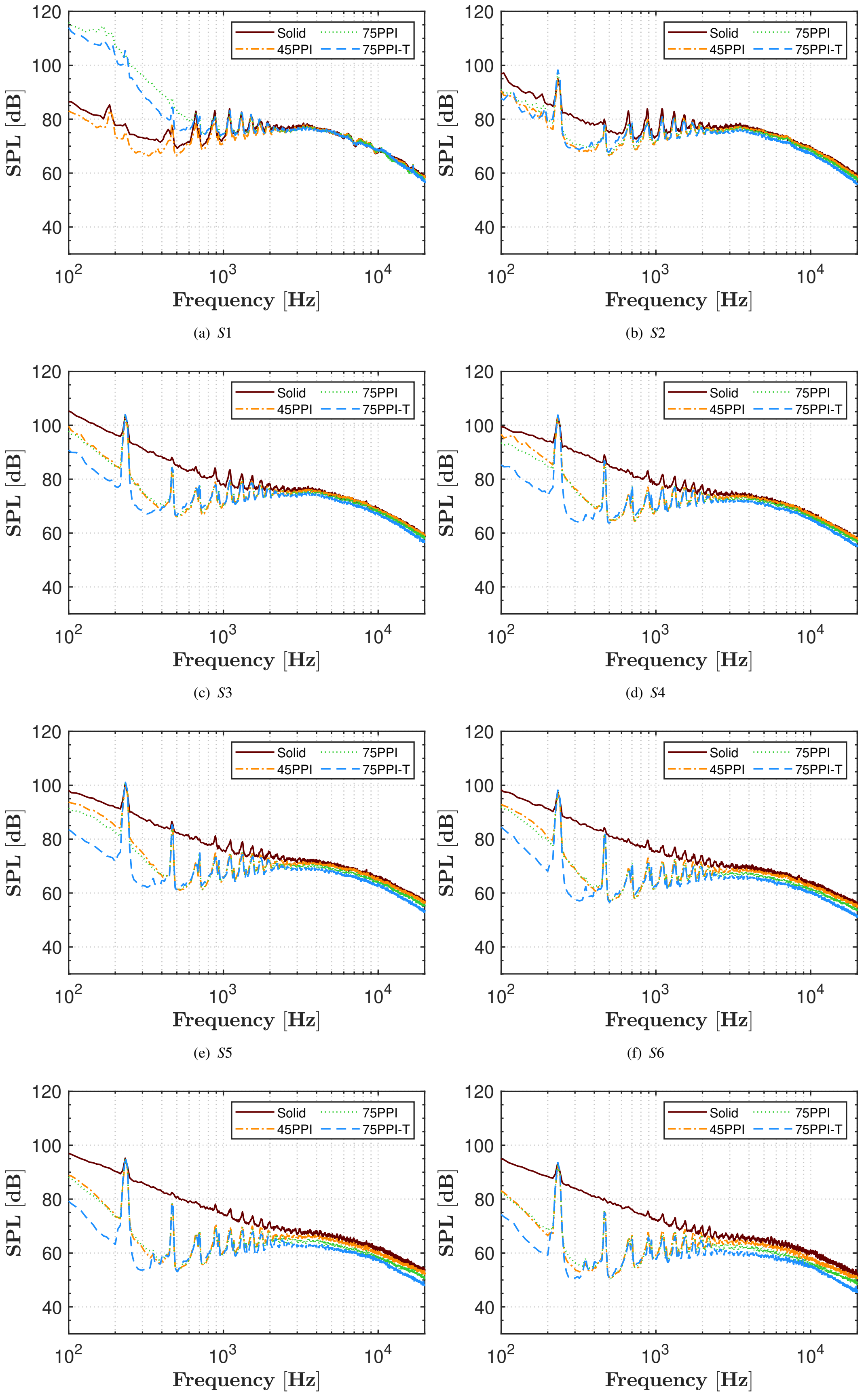

(a) $S1$ (b) $S2$ (c) $S3$ (d) $S4$ (e) $S5$ (f) $S6$ (g) $S7$ (h) $S8$

Figure S7. Near-field SPL of propeller operating at 7000 RPM, across $S1-S8$ microphone positions subjected to ground proximity of $L/R = 1.0$.

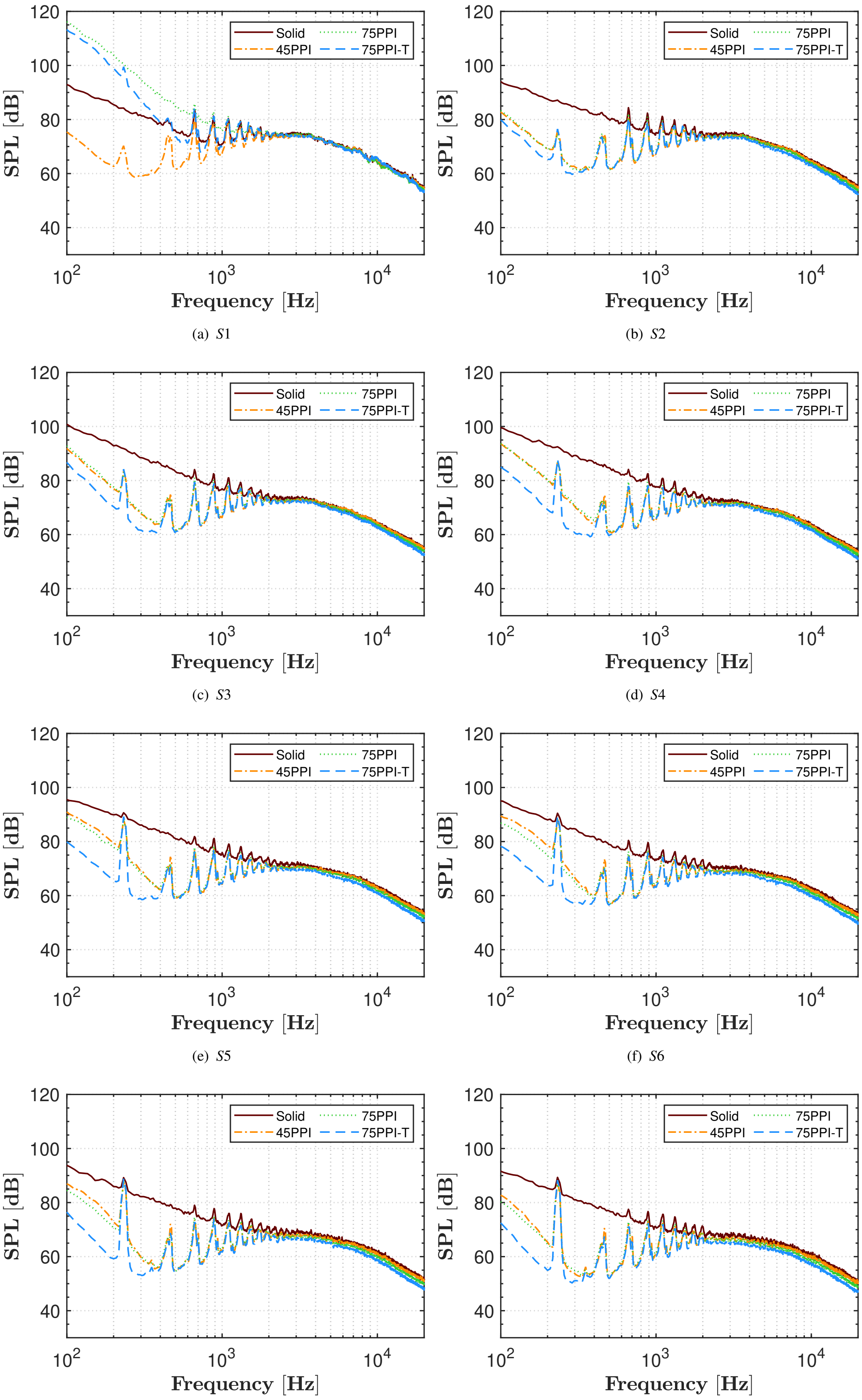

(a) $S1$ (b) $S2$ (c) $S3$ (d) $S4$ (e) $S5$ (f) $S6$ (g) $S7$ (h) $S8$

Figure S8. Near-field SPL of propeller operating at 7000 RPM, across $S1-S8$ microphone positions subjected to ground proximity of $L/R=2.0$.

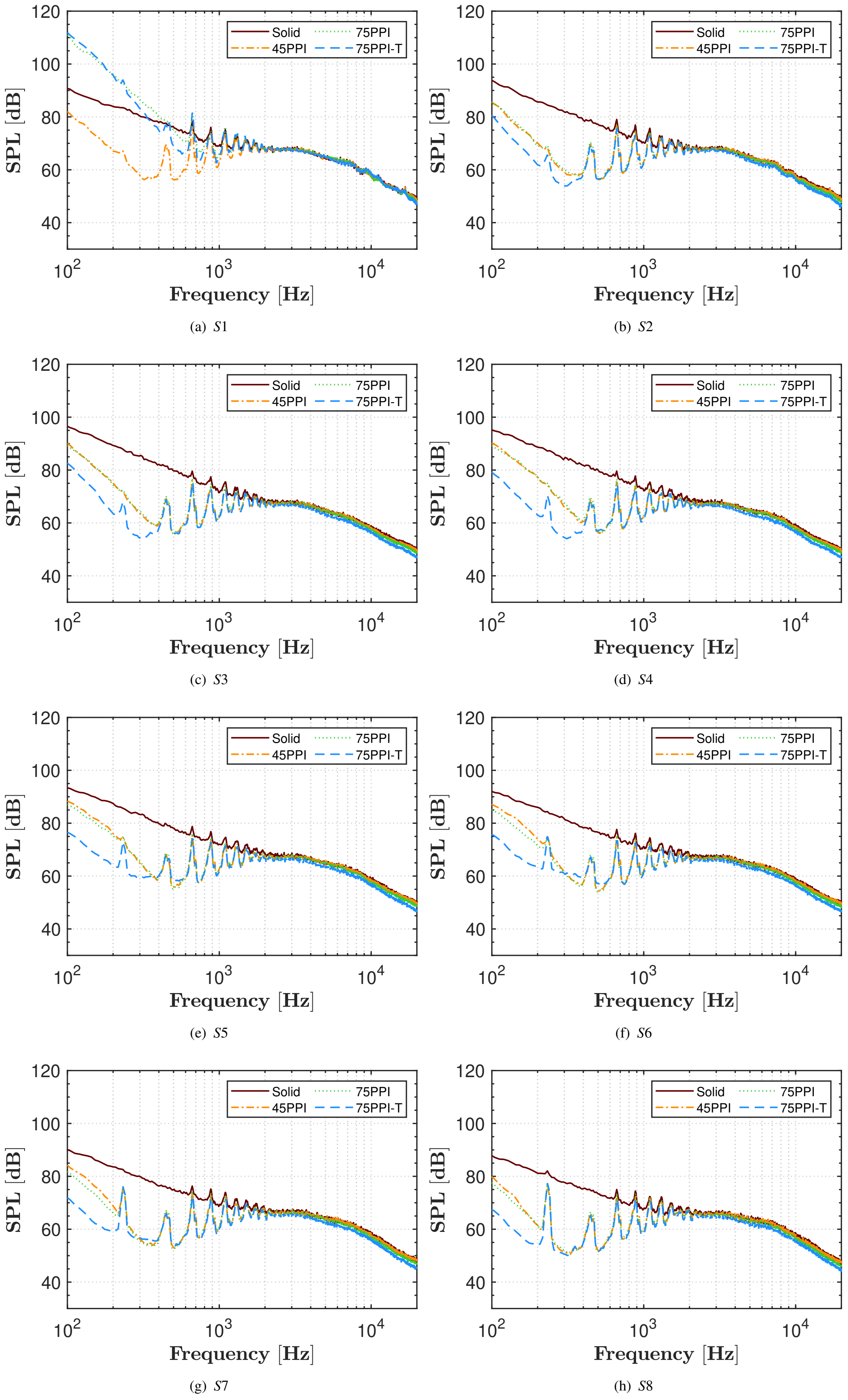

Figure S9. Near-field SPL of propeller operating at 7000 RPM, across $S1-S8$ microphone positions subjected to ground proximity of $L/R = 4.0$.
